# Perceived Environmental Corporate Social Responsibility and Employees’ Innovative Behavior: A Stimulus–Organism–Response Perspective

**DOI:** 10.3389/fpsyg.2021.777657

**Published:** 2022-01-31

**Authors:** Weiwei Wu, Li Yu, Haiyan Li, Tianyi Zhang

**Affiliations:** ^1^School of Management, Harbin Institute of Technology, Harbin, China; ^2^Business School, Shantou University, Shantou, China; ^3^Research Center of “Chaoshang” Innovation and Entrepreneurship, Shantou University, Shantou, China

**Keywords:** environmental corporate social responsibility, employees’ innovative behavior, organizational identification, organizational trust, S-O-R model

## Abstract

Drawing from the stimulus-organism-response (S-O-R) model, this study examines how and under what circumstances perceived environmental corporate social responsibility (ECSR) affects innovative behavior of employees in the context of environmental protection. Using a sample of 398 employees from different firms in the high energy-consuming industry of China, the results indicate that, at first, perceived ECSR provides a positive effect on organizational identification. Secondly, organizational identification has a positive influence on the innovative behavior of employees. Thirdly, organizational identification plays an important mediating effect between perceived ECSR and the innovative behavior of employees. Fourthly, both the effect of perceived ECSR on organizational identification and the indirect effect of perceived ECSR on the innovative behavior of the employees *via* organizational identification will be stronger when the levels of organizational trust are high. These findings add new insights into the perceived ECSR-employees’ innovative behavior relationship and provide important managerial implications for enhancing ECSR perception to improve the innovative behavior of employees.

## Introduction

There is now considerable agreement that the activities of the firms are the main cause of environmental degradation (Tian and Robertson, 2019). In China, firms, especially those in the high energy-consuming industry, are at the heart of persistent debates around whether they have enough respect for the natural environment (Li et al., 2017). Controlling pollutant emissions from high energy-consuming firms and developing cleaner energy sources have become the core requirements of the economic construction of China (Zhang and Liu, 2019; Han, 2021). In such a context, firms that want to meet these requirements and survive need to depend more on innovation (Tang et al., 2021; Wang et al., 2021). As is widely accepted, employee innovation is the foundation of the innovation of firms (Shin et al., 2017; Liu et al., 2019). Existing research has suggested that innovative behavior of employees is extremely important not only because it can play a key role in the sustainable development of firms, but also helps their firms gain competitive advantages in the rising pressure associated with environmental protection (Galbreath, 2019; Javed et al., 2020). Therefore, it is worth exploring how to effectively promote the innovative behavior of employees at the present stage. The innovative behavior of employees is defined as a series of positive behavioral responses that employees recognize, generate new ideas for products, services, and implement new ideas (Scott and Bruce, 1994; Kwon and Kim, 2020; Yuan, 2021). Many studies on the effects of organizational-level factors on the innovative behavior of employees are mainly from the economic perspective, including work process-related lead userness (e.g., Wu C.-H. et al., 2020), public service motivation (e.g., Miao et al., 2018), high-performance work practices (e.g., Farrukh et al., 2021), and perceived innovation job requirement (e.g., Shin et al., 2017), but a strong theoretical understanding from the non-economic perspective remains lacking.

In the high energy-consuming industry, environmental corporate social responsibility (ECSR) activity of firms is often presented as a non-economic activity (Roeck and Delobbe, 2012). ECSR is described as a voluntarily environmental behavior that aims to mitigate the influence of firms on the natural environment (Rahman and Post, 2012). ECSR can reflect the efforts of firms in a kind of environmental protection activities, such as waste emission reduction, pollution reduction, and product recycling (Flammer, 2013; Shah et al., 2021; Zhang and Ouyang, 2021). In addition, with the rapidly growing environmental awareness in employees (Ahmed et al., 2020), employees are more likely to have a passion for challenging and creative tasks related to the environmental activities of the firms (Hur et al., 2018). In this context, when employees perceive that their firms are responsible for the natural environment, they are more likely to offer their new ideas to the overall ECSR program of the organization and put such new ideas into implementation. Previous research has indicated that ECSR, as an issue of concern to employees within firms, has increasingly been valued by firms as one environmental stimulus to elicit the behavioral responses of employees (Orazalin, 2020), which help firms obtain the attention and support of employees (Su and Swanson, 2019). Thus, it is worth exploring whether perceptions of employees toward ECSR activities of firms positively affect the innovative behavior of employees.

However, the internal mechanisms in the relationship between perceived ECSR and the innovative behavior of employees also remain unclear. Some studies have shown that perceived corporate social responsibility (CSR) may influence the organizational identification of employees (Cheema et al., 2020), while others discovered that individual identification is an important factor that could impact employee innovations (Litchfield et al., 2018). Considering organizational identification as a cognitive process, scholars have investigated the mediating effect of organizational identification in the relationship between individual perception and behavior (Tian and Robertson, 2019; Van Dick et al., 2020). As such, organizational identification might act as the role of a bridge in the relationship between perceived ECSR and employee innovation. Although prior research has indicated that a direct relationship exists between the perceptions of employees on CSR and employee innovation (e.g., Hur et al., 2018), the internal mechanisms in the relationship between perceived ECSR and the innovative behavior of employees are rarely known. Hence, our work focuses on the mediating role of organizational identification, which enables us to penetrate internal mechanisms in perceived ECSR - employees’ innovative behavior relationship.

Moreover, the boundary conditions of the relationship between perceived ECSR and the innovative behavior of employees have also not been fully explored by researchers. Previous research has suggested that the direct effect of the perceptions of employees on CSR on employee creativity was significant (Brammer et al., 2015), but others pointed out that perceived CSR has no direct impact on employee creativity (Kim et al., 2021). The reason for such inconsistencies is that scholars may ignore the influence of moderating factors on CSR perception – employee innovation relationship. Some scholars have pointed out that the perceptions of employees to firms’ behaviors are shaped by the level of organizational trust (Taniguchi and Marshall, 2018). Organizational trust is an important element in a work environment that creates a collaborative environment by giving employees a feeling of integrity, commitment, and dependence (Chathoth et al., 2011; Bak, 2020). As a concept that describes the extent to the trust of employees in the organization (Chathoth et al., 2011), organizational trust can inevitably strengthen or weaken the degree to which the ECSR affects the attitudes and behaviors of employees (Alfes et al., 2012). Therefore, the influence of organizational trust should be considered in our research framework to investigate the perceived ECSR - organizational identification - employees’ innovative behavior of relationship.

Accordingly, using the stimulus-organism-response (S-O-R) model, we examine the relationship between perceived ECSR as a stimulus and the innovative behavior of employees as the response, and the mediating role of organizational identification (organism) in perceived ECSR-employees’ innovative behavior relationship, and the moderating role of organizational trust in the relationship between perceived ECSR and the innovative behavior of employees. The S-O-R model originated from the field of behavioral psychology and is widely applied in the consumer behavior literature and organizational behavior literature (Ahmed et al., 2020; Huang et al., 2021). The S-O-R model is used to analyze how environmental stimulus effectively affects internal state of an individual, and then elicits individual behavior (Mehrabian and Russell, 1974; Jang and Namkung, 2009; Xu and Wang, 2019). The objectives of this study are threefold: First, we examine how perceived ECSR as a stimulus affects the innovative behavior of employees as a response by using the extended S-O-R model in the context of environmental protection. Second, organizational identification as the mediating role through which perceived ECSR affects the innovative behavior of employees, further offers new insight into how the perceptions of employees on implemented ECSR of firms affect the responses of employees. Finally, by assessing how organizational trust positively enhances the direct effect of perceived ECSR on organizational identification and strengthens the indirect effect of perceived ECSR on the innovative behavior of employees through organizational identification, we identify a potential boundary condition to these relationships, and thus, reveal under what circumstances employees are more (or less) motivated to improve their innovative behavior. This study tests these hypotheses based on a dataset of 398 employees from different firms in high energy-consuming industries of China. At present, firms in China are often related to social negligence and environmental pollution in the eyes of the public (Wei et al., 2017; Xu et al., 2018; Tian and Robertson, 2019; Li et al., 2020). Moreover, there is a need to fill knowledge gaps in the relationship between perceived environmental corporate social responsibility (ECSR) and the innovative behavior of Chinese employees from the high energy-consuming industry. Hence, China provides a suitable context to investigate these relationships among perceived ECSR, organizational identification, organizational trust, and innovative behavior of employees.

Our study makes three contributions: first, this study on the effects of perceived ECSR on the innovative behavior of employees will contribute to enriching the predictors of innovative behavior literature by identifying another organizational means of promoting the innovative behavior of employees. Although previous studies have suggested that CSR perception may be an important predictor for the innovation of employees (Hur et al., 2018), the role of perceived ECSR as a key antecedent to the innovative behavior of employees remains unclear. In addition, understanding that the theoretical connection between the perceived ECSR and the innovative behavior of employees from the stimulus-organism-response perspective can provide different effective methods to reduce the pressure of environmental protection for firms in China. Second, this study highlights the role of organizational identification in the enactment of innovative behavior. Although prior studies have suggested that individual identification has a positive effect on innovative behavior (Litchfield et al., 2018), scholars have not fully explored the role of organizational identification in the relationship between the perceived ECSR and the innovative behavior of employees. Based on the S-O-R model, this study expands the work in previous studies by examining the mediating effect of organizational identification in perceived ECSR- employees’ innovative behavior relationship. Finally, this study contributes to extending the boundary conditions of the innovative behavior of employees from the perspective of organizational trust. Previous studies have rarely explored under what circumstances perceived ECSR can effectively promote employee innovation (Hur et al., 2018).

## Research Background and Hypotheses Development

### Stimulus-Organism-Response Model

Based on the stimulus-response theory, Mehrabian and Russell (1974) posited the S-O-R model which states that environmental stimulus impacts the internal state of an individual, and then influences approach behaviors or averting behaviors of an individual. The stimulus refers to environmental factors that can be conceptualized as stimulating individuals and impacting their internal state in the S-O-R model (Eroglu et al., 2001). According to the research by Jacoby (2002), the environmental factors include everything we usually understand as external stimuli, such as perceived quality (product, atmospherics, and service), brand image, reputation, policy, and countless other influencing factors (Jang and Namkung, 2009; Kim and Lennon, 2013; Tang et al., 2019). The organism is considered to be an internal process which plays an intervening role in the relationship between the stimulus and the response emitted by an individual (Mehrabian and Russell, 1974; Jang and Namkung, 2009; Bigne et al., 2020). Besides, the response is regarded as the final outcomes that can be approached or averting behavior. Approach behavior is a positive action in a particular setting, yet averting behavior is an opposite behavior (Mehrabian and Russell, 1974).

**FIGURE 1:**
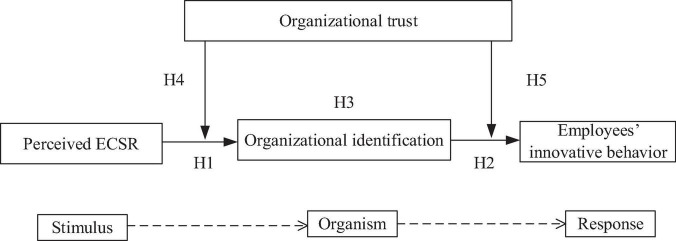
Proposed theoretical framework and hypotheses.

The S-O-R model provides an explanatory perspective on the innovative behavior of employees with regard to environmental effects (Xu and Wang, 2019). This model states that when an organism is stimulated by environmental factors, its internal processes, including its cognitive response (Ferdous et al., 2021), will change, resulting in it approaching or avoiding the environment that provides the stimulation. Organizational identification is a cognitive process that can be viewed as cognitive episodes (Jang and Namkung, 2009). The cognitive nature is regarded as “the mental structures and the processes involved in thinking about, understanding, and interpreting the stimuli and events of the environment” (Sánchez et al., 2006, p. 395). Therefore, organizational identification mediates the impacts of environmental factors on behaviors of employees. Under the setting of environmental protection, the stimuli consist of perceived ECSR. The internal psychological states of the organism include employee identification and other internal responses (e.g., emotional response; Jani and Han, 2015) that could elicit the behavioral responses of employees, including innovative behavior. Accordingly, we adopt the S-O-R model to examine the relationship among perceived ECSR, organizational identification, and innovative behavior of employees. Furthermore, previous studies have investigated that organizational trust plays a key role in improving perceptions of individuals and promoting positive workplace attitudes, such as perceived HRM practices (Alfes et al., 2012) and job satisfaction (Lee et al., 2013). Organizational trust describes the extent to which employees believe their organization (Chathoth et al., 2011). Organizational trust is regarded as an important element in a work environment and creates a collaborative environment by giving employees a feeling of integrity, commitment, and dependence (Chathoth et al., 2011; Ertürk and Vurgun, 2015). According to the S-O-R model, when perceived ECSR- organizational identification - employees’ innovative behavior relationship is considered as a stimulus-organism-response relationship, organizational trust might affect this relationship by creating a collaborative environment. Thus, we introduce organizational trust as the moderator into our extended S-O-R model (presented in Figure 1).

### Perceived Environmental Corporate Social Responsibility and Employee Responses

Environmental corporate social responsibility (ECSR) is from the notions of environmental management and CSR (Chuang and Huang, 2018). ECSR plays a vital part in the process of the impact of the activities of firms on the natural environment (Kim et al., 2017; Shah et al., 2021). According to Baughn et al. (2007), American enterprises have higher levels of CSR compared to other countries, but the ECSR level of American enterprises is lower. As such, high CSR does not always produce high ECSR (Chuang and Huang, 2018). Mazurkiewicz (2004) has defined ECSR as “the duty to cover the environmental implications of the operations, products, and facilities; elimination of waste and emissions; maximization of the efficiency and productivity of its resources; and minimization practices of the company that might adversely affect the enjoyment of the resources of the country by future generations.” According to the extant definitions of ECSR and the purpose of our study, we defined perceived ECSR as the subjective perception that employees perceive the extent to which the ECSRs of organizations are to be fulfilled and to evaluate his/her organization.

To date, however, the vast majority of studies in the ECSR literature mainly paid attention to the organizational level of analysis (Cordeiro and Tewari, 2014; Forcadell et al., 2021; Rela et al., 2021). For instance, studies on the organizational level have examined the effect of ECSR on organizational performance, such as financial performance (Lioui and Sharma, 2012; Zhang and Ouyang, 2021), export performance (Xu et al., 2018), innovation performance (Wu W. et al., 2020), business competitiveness, and environmental performance (Chuang and Huang, 2018; Orazalin, 2020). Recently, studies on the ECSR literature have begun to focus on the individual-level analysis of the effect of perceived ECSR on responses (Hur et al., 2018; Su and Swanson, 2019). Within the individual-level ECSR literature, it is a large number of studies on how the ECSR activities of a firm affect the responses of employees, such as trust and pride (Roeck and Delobbe, 2012; Su and Swanson, 2019), organizational commitment and identification (Hofman and Newman, 2014; Kim et al., 2016; Zhou et al., 2018), empathy (Tian and Robertson, 2019), and job satisfaction of employees (Ilkhanizadeh and Karatepe, 2017; Kim et al., 2018). Another stream of the individual level of ECSR literature has also shown how ECSR perception affects the behavioral responses of employees (Ruepert et al., 2017; Ahmad et al., 2021). For example, many researchers have reported that employees who positively perceive the ECSR activities of the firms are more likely to make a kind of the behavioral response of employees, such as employee creativity (Hur et al., 2018) and organizational citizenship behavior (Cheema et al., 2020).

### Perceived Environmental Corporate Social Responsibility and Organizational Identification

Organizational identification refers to “the degree to which a member defines him or herself by the same attributes that he or she believes define the organization” (Dutton et al., 1994; Roeck and Farooq, 2017). The psychological process of organizational identification explains internal processes that intervene between external stimulus to the establishment or maintenance of a relationship with their social groups of reference and attitudes of individuals (Dutton et al., 1994; Roeck and Farooq, 2017). In the context of environmental protection, the ECSR perception of employees can serve as a stimulus that affects the attitudes of employees (Ilkhanizadeh and Karatepe, 2017). Perceived ECSR focuses on the evaluation and understanding of individuals on the environmental responsibility of the organization in various aspects (Parker et al., 2003; Turker, 2009).

According to Kim et al. (2010), Korschun et al. (2014), and Afsar et al. (2018), employees who are attracted by the organizational image from the effort of environment protection are more likely to identify with environmentally responsible firms. This suggests that employees who are impacted by image evaluation of the organization are especially sensitive to the ECSR activities of their firm (Vlachos et al., 2010; Lee et al., 2013; Farrington et al., 2017) because ECSR can reflect the image of firms whether a firm strives to protect the natural environment (Rahman and Post, 2012). The other way round, the ECSR perceptions of employees impact the attractiveness of image of their organization because it contributes to increasing the consistency between values of employees and organizational values (Kim et al., 2010; Glavas, 2016; Afsar et al., 2018). According to Dutton et al. (1994), organizational identification of their organization is reinforced when employees believe to have the same attributes (e.g., values, beliefs, and goals) with the organization. Supporting these theoretical arguments, firms positively engaged in ECSR activities tend to enhance the image so that employees are more likely to increase organizational identification of the employee for the company (De Roeck et al., 2016; Islam et al., 2016; Afsar et al., 2018; Su and Swanson, 2019; Cheema et al., 2020). Thus, the perception of employees of ECSR activities of their firms may positively impact the degree of their organizational identification. In association with the literature above, we proposed the following hypothesis:

***Hypothesis1.*** *Perceived ECSR has a positive effect on organizational identification.*

### Organizational Identification and Innovative Behavior of Employees

Existing studies have suggested that organizational identification is related to the behavioral responses of individuals toward their firms (e.g., Dukerich et al., 2002; Madjar et al., 2011). Employees tend to integrate organizational values, goals, and beliefs into the belief categories related to themselves (Dutton et al., 1994; Roeck and Farooq, 2017), and then adopt positive behavioral responses consistent with their values, goals, and beliefs (Ashforth et al., 2008; Madjar et al., 2011; Barba-Sánchez and Atienza-Sahuquillo, 2017).

The innovative behavior of employees represents a type of individual behavioral response in the S-O-R model and is defined as a series of behaviors that employees recognize, generate new ideas for products and services, and implement new ideas (Scott and Bruce, 1994), and is consistent with the organizational values, beliefs, and goals (Dutton et al., 1994; Roeck and Farooq, 2017). Previous research has provided support in that when employees identify with their organization, they will positively vest in the success and survival of the firm and are motivated to adopt positive behavioral responses of individuals (Ashforth and Mael, 1989; Song et al., 2019). Thus, this study states that organizational identification may be a crucial factor that affects the innovative behavior of employees. More specifically, our study argues that employees who identify with a firm because of the same values, goals, and beliefs with organizations are more likely to support their firms (Ashforth et al., 2008), which may positively promote the behavioral responses of employees (e.g., innovative behavior of employees) that support their firms, such as generating new ideas and securing all resources to implement these novel and useful ideas. Thus, we hypothesize:

***Hypothesis2***. *Organizational identification has a positive effect on the innovative behavior of employees.*

### The Mediating Role of Organizational Identification

The pattern of relationships discussed above indicates the potential impacts of ECSR, such that perceived ECSR as a stimulus may indirectly affect the innovative behavior of employees (i.e., behavioral responses of individuals) through organizational identification. Consistent with the S-O-R model, some studies indicate that perceived ECSR is regarded as a vital environmental stimulus factor that can impact the degree of identification of employees, and then the propensity of employees to produce different behavioral responses in the organization (Castro-Gonzalez et al., 2019; Boan and Dedeolu, 2020; Cheema et al., 2020; Shah et al., 2020). For instance, Tian and Robertson (2019) confirmed that perceived CSR (include environmentally responsible practices; Turker, 2009) could influence tendency of employees to identify with the firm and then influence behavioral response of employees to participate in supporting firm. They also proved that perceived CSR could indirectly influence the behavioral responses of individuals *via* organizational identification (Brammer et al., 2015). As a result, organizational identification is considered as an important organism that plays an intervening role in the relationship between perceived ECSR (i.e., stimulus) and innovative behavior of employees.

In the S-O-R model, the organizational identification of employees as the organism is related to perceived ECSR and innovative behavior of employees. More specifically, our study suggests that employees tend to identify with their company when they regard their company as an organization responsible for the environment (i.e., ECSR; Turker, 2009), and therefore may be motivated to generate new ideas for products, services, and implement new ideas that support their firms (Xu and Wang, 2019). In particular, employees who identify with their company because of its environmental responsibility tend to support the ECSR activities of the company by fostering innovative behavior (Madjar et al., 2011; Wu W. et al., 2020). Taken together, we suggest that organizational identification of employees, as a mediator, enhances the positive effect of perceived ECSR on the innovative behavior of employees. Therefore, this study proposed the following hypothesis:

***Hypothesis3.*** *Organizational identification positively mediates the relationship between ECSR and innovative behavior of employees.*

### The Moderating Role of Organizational Trust

#### Perceived Environmental Corporate Social Responsibility, Organizational Trust, and Organizational Identification

Organizational trust is conceptualized as positive expectations of employees for the intentions and behaviors of multiple organizational members based on organizational roles, relationships, experiences, and interdependencies (Chathoth et al., 2011). The organizational trust consists of integrity, commitment, and dependence (Chathoth et al., 2011). Integrity refers to the principles and values that the trustee adheres to and accepted by the trustor, while commitment is about “a sense of loyalty in the action of the individual leading to identification and association with a given organization” (Chathoth et al., 2011). Dependability captures factors that relate to the loyalty of the organization to its employees and is considered as the degree of credibility of employees to the organization (Chathoth et al., 2011). Studies on the literature state that organizational trust is considered to be a critical variable that affects organizational effectiveness (Ertürk and Vurgun, 2015). In the context of environmental protection, organizational trust represents the level of positive expectation that employees perceive toward a voluntarily environmental behavior of firms and the degree to which they believe what firms show the efforts of firms in a kind of environmental protection activities (Hosmer, 1995). It suggests that employees with high levels of organizational trust are those who tend to have positive expectations about organizational activities.

Recently, it has been argued that high organizational trust affects the relationships between the perception of employees of voluntarily environmental behaviors and the organizational identification of firms (Dirks and Ferrin, 2002; Farooq et al., 2014, 2017). By doing so, a high level of organizational trust is more likely to strengthen the effect of perceived ECSR on organizational identification. In addition, high organizational trust can also make it easier for employees to perceive the environmental responsibility of firms, bolstering the positive effect of perceived ECSR on organizational identification. In contrast, employees with low levels of organizational trust do not react strongly to the image of the organization (Perry and Mankin, 2007). Specifically, when employees are at a low level of organizational trust, they have low expectations for any activity of the organization because these employees have questioned the integrity and commitment of the organization and reduced their dependence on the organization (Thomas, 2015). As such, low organizational trust is less likely to enhance the organizational identification that employees may experience under ECSR perception. According to the above studies, we add organizational trust as a moderator in the S-O-R model, we hypothesize:

***Hypothesis4.*** *Organizational trust positively moderates the relationship between perceived ECSR and organizational identification, such that the positive relationship is stronger for employees with higher levels of organizational trust.*

#### Perceived Environmental Corporate Social Responsibility, Organizational Trust, and Innovative Behavior of Employees

Organizational trust is viewed as a critical moderating variable that impacts employee behavior (Ertürk, 2010; Su and Swanson, 2019; Bak, 2020) because it seems to provide more insights into employee behavior based on organizational environment. In an organizational setting, a high level of organizational trust positively strengthens the impact of organizational identification on the behaviors of employees (Ertürk, 2010). For instance, employees with high levels of organizational trust are more likely to identify with the focus of organizational activities on improving the quality of the environment where engaging in innovative behaviors in response to organizational environmentally responsible is expected (Brammer et al., 2015; Hur et al., 2018).

It follows that the employees with a high level of organizational trust will be positively motivated to generate new ideas in response to organizational activities they perceive at work but also encourage them to engage in innovative behavior by implementing such ideas for the success and survival of firms (Hansen et al., 2011; Alfes et al., 2012; Lee et al., 2013). Specifically, when employees perceive the image of the organization, that the firms will be responsible for the quality of the environment, employees with high levels of organizational trust will positively identify with their firms because of their ECSR perceptions, and they are more inclined to contribute their new ideas and secure all resources to implement new ideas that support the values, goals, and beliefs of the firm. Conversely, employees with low levels of organizational trust will not be impacted by the image of organization (Pučėtaitė and Lämsä, 2008), such employees are less likely to respond positively to their organizational activities (Archimi et al., 2018). As such, even if employees with low levels of organizational trust identify with their organization due to the ECSR perceptions, the indirect effect of perceived ECSR on the innovative behavior of employees will be weaker. Altogether, we proposed the following hypothesis:

***Hypothesis5.*** *Organizational trust positively moderates the indirect effect of perceived ECSR on innovative behavior of employees via organizational identification, such that the indirect effect will be stronger when the levels of organizational trust are high.*

## Methodology

### Sample and Data Collection

To test all hypotheses of the current study, we collected data from employees of firms in the high energy-consuming industry of China. The survey was conducted from April to July 2020. According to “National Bureau of Statistics of the People’s Republic of China (2011)” issued by the Chinese government (National Bureau of Statistics), high energy-consuming industries mainly include power, steel and instrument manufacturing, petrochemicals and chemicals, non-ferrous metals, pharmaceuticals, paper, coal, building materials, textiles, and mining. Because a list of firms with the telephone numbers and e-mail addresses of employees has not been fully disclosed in China, many studies obtain such lists through government agencies (Walker et al., 2014; Wei et al., 2017). Thus, we approached the government agencies and got a list of high energy-consuming firms with the contact information of Human Resource (HR) managers. The high energy-consuming firms in this list are more than 14,000 firms, from which we randomly selected 500.

To recruit employees as participants, we first discussed the objectives and procedures of our study with HR managers of firms. The HR managers randomly selected employees and provided us with a list of 500 participants. The survey participants mainly included managers, technical staff, and production personnel involved in innovation activities of their firms. Then, the researchers sent recruitment emails to all participants before the investigation, informing them of the academic purpose of this survey. We have promised them that the questions they answered are confidential and only used for academic research. We changed the order of predictor variable (perceived ECSR), mediating variable (organizational identification), moderating variable (organizational trust), and control variables in our questionnaire. Next, we asked participants to answer all survey questions. Based on the feedback of participants, we confirmed that all items included in the survey were clear and comprehensive.

The researchers conducted a questionnaire survey after obtaining the consent of all participants included in the study. Data were collected in two stages: in the first stage, participants completed Questionnaire A regarding perceived ECSR, organizational trust, and control variables (gender, age, education, industry, and tenure of employees). After 1 month, in the second stage, participants were asked to complete Questionnaire B on organizational identification and innovative behavior of employees. During the questionnaire survey, the researchers asked managers, technical staff, and production personnel to complete the questionnaire survey. We conducted a Kruskal-Wallis H to test the position distributions of the respondents (*x*^2^ = 2.793, *Asymp*.*Sig*. = 0.425 > 0.05). The results revealed that there is no significant difference in their positions.

According to Comrey (1988), a sample size of below 100 is not suitable for factor analysis, a sample size of about 200 is good for ordinary factor-analytic work, and a sample size of over 300 is great. We sent a total of 500 questionnaires to employees working in high energy-consuming industries (e.g., non-ferrous metals) in China. After excluding missing data and those failing to meet the questionnaire requirements, our final sample consists of 398 employees, representing an overall response rate of 79.60%. The final sample displays about 52.76% of employees were male. Of the 398 responding employees, 16.08% held board senior managers, middle managers, and general managers, and 33.17% were technical staff, 28.64% were production personnel, and 22.11% were others. Most employees were under 30 years old, 13.57% of organizational tenure of employees have been employed for 11–20 years, 5.28% have been employed for more than 20 years within the firm, and 53.02% of the education of employees were bachelor’s degrees.

### Measures

Walker et al. (2014) pointed out that due to the lack of public data in China, academic research often needs to rely on surveys to collect the data required by the research. Our survey is based on face-to-face interviews and previous research. According to the method of back translation, all items were translated into Chinese (Reynolds et al., 1993). All items were measured on a 5-point Likert scale ranging from 1 (*strongly disagree*) to 5 (*strongly agree*) and depicted in Table 1.

**TABLE 1:** Measurement items.

Variables	Items	Factor loading	CR	AVE	Cronbach’s alpha
Perceived ECSR	PECSR1: “I can feel our company implements special programs to minimize its negative impact on the natural environment.”	0.804	0.923	0.665	0.922
	PECSR2: “I can feel our company participates in activities which aim to protect and improve the quality of the natural environment.”	0.829			
	PECSR3: “I can feel our company has the necessary equipment to reduce its negative environmental impact.”	0.817			
	PECSR4: “I can feel our company makes well-planned investments to avoid environmental degradation.”	0.825			
	PECSR5: “I can feel our company targets sustainable growth which considers future generations.”	0.813			
	PECSR6: “I can feel our company makes investment to create a better life for future generations.”	0.805			
Organizational identification	O11: “Our company’s successes are my successes”	0.791	0.917	0.647	0.916
	O12: “When I talk about our company, I usually say we rather than they.”	0.776			
	O13: “When someone criticizes our company, it feels like a personal insult.”	0.826			
	O14: “I am very interested in what others think about our company.”	0.793			
	O15: “When someone praises our company, it feels like a personal compliment.”	0.842			
	O16: “If a story in the media criticized our company, I would feel embarrassed.”	0.797			
Organizational trust	OT1: “Our company treats me fairly and properly.”	0.767	0.908	0.663	0.907
	OT2: “Our company communicates with me openly and honestly.”	0.855			
	OT3: “Our company tells me everything that I want to know.”	0.833			
	OT4: “Our company considers my advice valuable.”	0.840			
	OT5: “Our company maintains a long-term relationship with me.”	0.772			
Employees’ innovative behavior	EIB1: “I can search out new technologies and new processes in work.”	0.831	0.941	0.725	0.940
	EIB2: “I often generate creative ideas in my work.”	0.877			
	EIB3: “I often promote and champion new ideas to others.”	0.852			
	EIB4: “I often investigate and secure founds needed to implement new ideas.”	0.824			
	EIB5: “I often develop adequate plans and schedules for the implementation of new ideas.”	0.872			
	EIB6: “Generally speaking, I am an innovative person.”	0.852			

*CR, composite reliability; AVE, average variance extracted.*

#### Perceived Environmental Corporate Social Responsibility

Based on the research of Turker (2009), we retained six items from the social and nonsocial dimension of stakeholders of the CSR scale to measure the perception of employees in that which employees perceived activities of their organization protect the natural environment (Roeck and Delobbe, 2012). A sample item is “I can feel our company implements special programs to minimize its negative impact on the natural environment.”

#### Organizational Identification

Based on the research of Mael and Ashforth (1992) and Brammer et al. (2015), our study adapts a measure of organizational identification on six items measuring employees’ degree of identification in an organization. Sample items include: “Our company’s successes are my successes” and “When I talk about our company, I usually say we rather than they.”

#### Organizational Trust

According to the work of Chathoth et al. (2011), we adapt a measure of organizational trust based on the five items that are used to evaluate degree of trust of employees in the organization. The five items are used to measure organizational trust appear in Table 1.

#### Innovative Behavior of Employees

Since the innovative behavior of employees has been conceptualized as a workplace behavior by Yuan and Woodman (2010) and Wu C.-H. et al., 2020, we measure the innovative behavior of employees based on a six-item scale from Scott and Bruce (1994). Sample items are “I can search out new technologies and new processes in work” and “I often generate creative ideas in my work.”

#### Control Variables

Past ECSR research implies that some demographic characteristics of employees, such as age, gender, education, and tenure, have been related to general workplace behaviors, which may impact the results of the hypothesized relationships in our study (Rahman and Post, 2012; Tian and Robertson, 2019). Therefore, we controlled for the gender, age, education, and organizational tenure (years) of employees in our analyses. The gender of employees was coded as “1” for males and “2” for females. Age of employees was coded as “1” for employees aged between 18 and 30, “2” for employees aged between 31 and 40, “3” for employees aged between 41 and 50, and “4” for employees aged 51 or above. The education of employees was coded as “1” for a high school education or below, “2” for college, “3” for a bachelor’s degree, and “4” for a master’s degree or above. Tenure was coded as “1” for 2 years or below, “2” for 3 to 5 years, “3” for 6 to 10 years, “4” for 11 to 20 years, and “5” for 21 years or above. In addition, this study controlled for industry, as this may affect innovative behavior of employees. This study surveys these employees from high energy-consuming companies in a variety of high energy-consuming industries, including power, steel and instrument manufacturing, petrochemicals and chemicals, non-ferrous metals, pharmaceuticals, paper, coal, building materials, textiles, and mining. The industry was coded as “1” for power, “2” for steel and instrument manufacturing, “3” for petrochemical and chemical, “4” for non-ferrous metals, “5” for pharmaceutical, “6” for paper, “7” for coal, “8” for building materials, “9” for textiles, “10” for mining, and “11” for other industries.

### Reliability and Validity Analysis

The KMO of perceived ECSR, organizational identification, organizational trust, and innovative behavior of employees were all over 0.70, the significance of Bartlett’s test is 0.000, and the cumulative variance contribution rate of common factors extracted by each variable is more than 70%, which reveals that it is suitable for factor analysis. We examined all the items using the exploratory factor analysis (EFA). By adopting the principal component analysis method, EFA was carried out for all items. The results showed that four factors were extracted: perceived ECSR, organizational identification, organizational trust, and innovative behavior of employees. In addition, the minimum standardized factor loading was 0.697,more than 0.5. Taken together, the four-factor structure was confirmed.

We tested the reliability and validity of our four variables *via* SPSS 21 and Amos 21. Cronbach’s alpha values of perceived ECSR, organizational identification, organizational trust, and innovative behavior of employees were greater than 0.70 (Table 1), indicating that all survey scales show good reliability. This study used confirmatory factor analysis (CFA) to test our model fit. The AVE values of all of the constructs are above 0.5, and the composite reliability (CR) of each variable is larger than 0.8 in Table 1, thereby suggesting that has a high convergent validity (Fornell and Larcker, 1981). Moreover, results show that the off-diagonal coefficients are less than the square root of AVE for each construct (see Table 3). Meanwhile, the results in Table 2 indicate that the four-factor model was significantly superior to other models. Thus, there is a good discrimination validity among the variables.

**TABLE 2:** Results of confirmatory factor analysis.

Model	x^2^	df	x^2^/d*f*	Δ*x*^2^	RMSEA	NFI	RFI	CFI	IFI	TLI	SRMR
1.Four-factor model	358.995	224	1.603	–	0.039	0.949	0.943	0.980	0.980	0.978	0.039
2.Three-factor model (OI & OT = 1 factor)	1015.342	227	4.473	656.347	0.094	0.857	0.840	0.885	0.885	0.871	0.075
3.Three-factor model (PECSR & OT = 1 factor)	1124.245	227	4.953	765.250	0.100	0.841	0.823	0.869	0.869	0.854	0.088
4.Three-factor model (OT & EIB = 1 factor)	1463.318	227	6.446	1104.323	0.117	0.793	0.770	0.819	0.820	0.798	0.144
5.Three-factor model (PECSR & OI = 1 factor)	1651.478	227	7.275	1292.483	0.126	0.767	0.740	0.791	0.792	0.767	0.124
6.Three-factor model (OI & EIB = 1 factor)	1711.826	227	7.541	1352.831	0.128	0.758	0.731	0.783	0.783	0.758	0.149
7.Three-factor model (PECSR & EIB = 1 factor)	1766.763	227	7.783	1407.768	0.131	0.750	0.722	0.774	0.775	0.749	0.146
8.Two-factor model (PECSR & EIB = 1 factor; OI & OT = 1 factor)	2391.665	229	10.444	2032.670	0.154	0.662	0.627	0.683	0.684	0.650	0.160
9.Two-factor model (PECSR & OT = 1 factor; OI & EIB = 1 factor)	2429.049	229	10.607	2070.054	0.156	0.657	0.621	0.678	0.679	0.644	0.168
10.Two-factor model (PECSR & OI = 1 factor; OT & EIB = 1 factor)	2718.791	229	11.872	2359.796	0.165	0.616	0.576	0.635	0.637	0.597	0.191
11.One-factor model	3433.044	230	14.926	3074.049	0.187	0.515	0.467	0.531	0.532	0.484	0.152

*N = 398. PECSR, perceived ECSR; OI, organizational identification; OT, organizational trust; EIB, innovative behavior of employees.*

**TABLE 3:** Descriptive statistics and correlations of variables.

Variables	Mean	SD	1	2	3	4	5	6	7	8	9
1.Perceived ECSR	3.857	1.065	**0.816**								
2.Organizational identification	3.984	0.967	0.417***	**0.804**							
3.Organizational trust	3.855	1.051	0.668***	0.615***	**0.814**						
4.Employees’ innovative behavior	3.809	1.045	0.320***	0.369***	0.355***	**0.852**					
5.Gender	1.472	0.500	–0.018	0.024	0.026	–0.030	–				
6.Age	1.807	0.984	0.087*	0.132***	0.113**	0.077	0.207***	–			
7.Education	2.666	0.893	–0.045	–0.003	−0.114**	0.001	0.055	−0.257***			
8.Industry	6.701	3.576	−0.119**	–0.022	–0.050	−0.091*	0.141***	−0.134***	0.059	–	
9.Tenure	2.309	1.220	0.113**	0.125**	0.094*	0.010	−0.161***	0.285***	−0.141***	−0.189***	–

N = 398, *p < 0.10, **p < 0.05, ***p < 0.01 (two-tailed test). Bold stands for the square root of AVE.

### Common Method Variance

As this study collected data using questionnaires, there might be a problem with the Common Method Variance (CMV) (Podsakoff et al., 2003). To reduce the issues related to common method bias (Spector, 1994), first, we changed the order of all variables in our questionnaire to reduce predictions of participants. Second, we set the answers to the questionnaire as anonymous and signed a confidentiality agreement with employees. Participants were able to answer the questions by their spontaneous opinions, as this study emphasized that there was no definite answer to these questions. For the statistical control, based on the single factor test (Harman, 1961), we used SPSS 21.0 to analyze all the data. A total of 73.73% of the total variance of item interpretation is more than 60%, and 42.80% of the total variance of the first-factor interpretation is less than 50% (Fuller et al., 2016). We further conduct confirmatory factor analysis (CFA) to test the possibility of CMV. These results corroborated that the four-factor model is in good agreement with the data (*x*^2^ = 358.995, *df* = 224, *x*^2^/*df* = 1.603, RMSEA = 0.039, NFI = 0.949, RFI = 0.943, CFI = 0.980, IFI = 0.980, TLI = 0.978, SRMR = 0.039) and was significantly superior to one factor model (*x*^2^ = 3433.044, *df* = 230, *x*^2^/*df* = 14.926, RMSEA = 0.187, NFI = 0.515, RFI = 0.467, CFI = 0.531, IFI = 0.532, TLI = 0.484, SRMR = 0.152). Thus, these precautions effectively prevent the problems that would occur in the data of our study due to common method bias.

## Results

### Descriptive Statistics

Table 3 reports the means, standard deviations, and correlations of variables. The variance inflation factors for perceived ECSR (1.806), organizational identification (1.608), and organizational trust (2.400) are below the cutoff of 10, indicating that multicollinearity is not a problem in the current study. As expected, perceived ECSR is significantly related to the innovative behavior of employees (*r* = 0.320, *p* < 0.01). Furthermore, the correlations are consistent with the mediation of this study. The results report that perceived ECSR is significantly associated with organizational identification (*r* = 0.417, *p* < 0.01), and organizational identification significantly affects the innovative behavior of employees (*r* = 0.369, *p* < 0.01). We also tested the control variables. Particularly, age (*r* = 0.132, *p* < 0.01) and tenure (*r* = 0.125, *p* < 0.05) are significantly related to organizational identification. Industry (*r* = 0.091, *p* < 0.1) is significantly related to the innovative behavior of employees. We found gender and education are not significantly related to organizational identification or innovative behavior of employees in Table 3. Organizational trust is significantly associated with organizational identification and innovative behavior of employees, which suggests that organizational trust may strengthen the effect of perceived ECSR on both organizational identification and innovative behavior of employees.

### Hypothesis Testing

This study adopted PROCESS macros (Hayes, 2013) to test all of our hypotheses. The bootstrapping procedure with 5,000 bootstrapped samples was employed to test these effects. If 95% confidence intervals (CI) do not include zero, the direct and indirect effects are significant. From Table 4, the results indicated that perceived ECSR affects positively organizational identification (*b* = 0.103, *p* < 0.01), thereby supporting Hypothesis 1. Meanwhile, education (*b* = 0.118, *p* < 0.01) has a positive effect on organizational identification. Results confirmed that organizational identification has a positive impact on the innovative behavior of employees (*b* = 0.372, *p* < 0.01). Therefore, Hypothesis 2 is supported. Additionally, this study tested the control variables: gender (b = −0.122, *p* > 0.05), age (b = −0.088, *p* > 0.05), education (b = 0.225, *p* < 0.01), industry (b = −0.017, *p* > 0.05), and tenure (b = −0.006, *p* > 0.05) and found only education to be significant.

**TABLE 4:** Hierarchical regression analysis results.

Variables	DV: Organizational identification			DV: Employees’ innovative behavior		
	*b*	SE	*t*	*b*	SE	*t*
**Predictors**						
Perceived ECSR	0.103*	0.051	2.033	0.096	0.060	1.597
Organizational identification				0.372**	0.068	5.498
Organizational trust	0.631**	0.049	12.789	0.185	0.072	2.572
Perceived ECSR × Organizational trust	0.148**	0.028	5.301			
Organizational identification × Organizational trust				0.171**	0.042	4.132
**Controls**						
Gender	0.005	0.079	0.059	−0.101	0.100	−1.008
Age	0.053	0.042	1.255	0.054	0.054	0.999
Education	0.118**	0.043	2.723	0.059	0.056	1.055
Industry	0.007	0.011	0.604	−0.022	0.014	−1.647
Organizational tenure	0.059	0.033	1.802	−0.066	0.042	−1.576
R^2^	0.434			0.221		
F-value	37.336**			12.195**		

*p < 0.05, **p < 0.01, Bootstrap sample: n = 5,000. SE, standard error.

Supporting Hypothesis 4, we found that the coefficient of the interaction involving perceived ECSR and organizational trust is positive and significant (b = 0.148, *p* < 0.01) in Table 4. As shown in Table 5, testing the effects on organizational identification at specific values (i.e., the mean and plus/minus one SD from mean) of organizational trust values indicated that the conditional direct effect of organizational trust values on organizational identification was significant at high levels of organizational trust [i.e., the mean plus one SD; conditional direct effect: b=0.258, p<0.01, CI [0.126, 0.391)] and medium levels of organizational trust (i.e., the mean; conditional direct effect:b=0.103, p<0.01, CI (0.003, 0.203)], but not at low levels of organizational trust [i.e., the mean minus one SD; conditional direct effect: b=0.05, p>0.05, CI (−0.147, 0.042)].

**TABLE 5:** Conditional effects of perceived environmental corporate social responsibility (ECSR) on organizational identification at values of organizational trust.

Organizational trust	Effect	SE	*t*	*p*	LLCI	ULCI
Low (M-1SD)	–0.052	0.048	–1.084	0.279	–0.147	0.042
M	0.103	0.051	2.033	0.043	0.003	0.203
High (M+1SD)	0.258	0.068	3.830	0.000	0.126	0.391

*Bootstrap sample: n = 5,000. SE, standard error. Values for organizational trust represent the mean and plus/minus one SD from mean.*

In Table 4, the coefficient of the interaction between organizational identification and organizational trust is significantly positive (b=0.171, p<0.01), which demonstrated the moderating positive effect of organizational trust on the link between organizational identification and innovative behavior of employees. Further, Table 6 displays the results of the indirect effect of the level of organizational trust. The findings suggest that perceived ECSR is indirectly and significantly related to the innovative behavior of employees through organizational identification for employees with high [i.e., the mean plus one SD; conditional indirect effect: b=0.143, p<0.05, CI (0.045, 0.282)], but not at medium [i.e., the mean; conditional indirect effect: b=0.038, p>0.05, CI (−0.005, 0.1)] and low [i.e., the mean minus one SD; conditional indirect effect: b=0.01, p>0.05, CI (−0.044, 0.017)] levels of organizational trust. Taken together, our findings support Hypothesis 3 and Hypothesis 5.

**TABLE 6:** Conditional indirect effect of perceived ECSR on employees’ innovative behavior through organizational identification moderated by organizational trust.

Dependent variable	Moderator: Organizational trust				
	Condition	Effect	BootSE	Boot 95% CI	
				LLCI	ULCI
Employees’ innovative behavior	Low (M-1SD)	–0.01	0.015	–0.044	0.017
	M	0.038	0.027	–0.005	0.100
	High (M+1SD)	0.143	0.061	0.045	0.282

*Bootstrap sample: n = 5,000. SE, standard error. Values for organizational trust represent the mean and plus/minus one SD from mean.*

**FIGURE 2:**
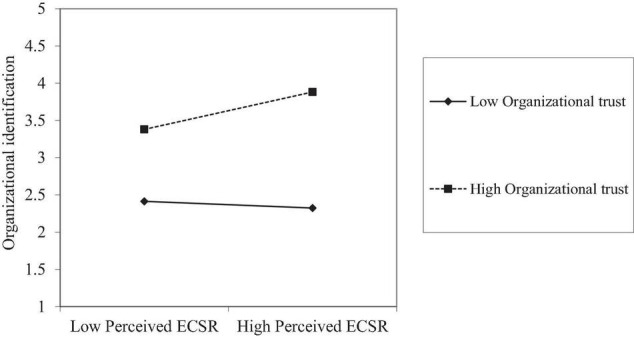
Interaction of perceived environmental corporate social responsibility (ECSR) and organizational trust on organizational identification.

**FIGURE 3:**
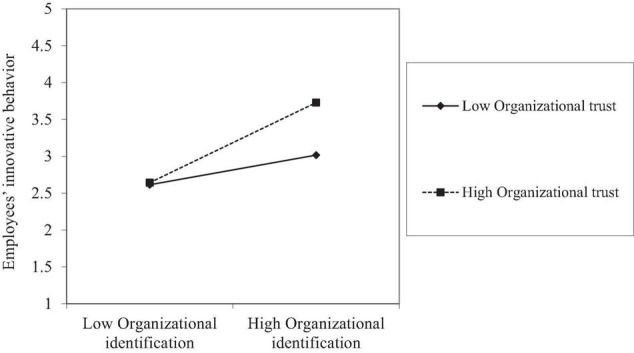
Interaction of organizational identification and organizational trust on the innovative behavior of employees.

Figures 2, 3 show when organizational trust value is high, as perceived ECSR increases, the increase in organizational identification and innovative behavior of employees is much steeper than under the condition of low organizational trust. It suggests that the effect of perceived ECSR on organizational identification and the indirect effect of perceived ECSR on the innovative behavior of employees *via* organizational identification became stronger when the level of organizational trust is higher.

## Discussion

Based on a sample of employees from high energy-consuming industries and drawing upon the S-O-R model, our findings suggest that employees who perceive their firms are responsible for the natural environment tend to identify with their organization, and in turn affect the innovative behavior of employees. Our findings also suggest that organizational trust moderates positively the link perceived ECSR and organizational identification, while organizational trust also positively moderates the strength of the positive indirect link between the perceived ECSR and the innovative behavior of employees through organizational identification. Thus, a few key theoretical contributions and managerial implications are made in this study.

### Theoretical Contributions

Our research contributes several theoretical insights. First, our first contribution is to the innovative behavior literature. This study extends the investigation of ECSR perception into the innovative behavior domain and identifies the predictive role of perceived ECSR on the innovative behavior of employees. Although previous studies have verified that perceived ECSR is a crucial predictor for the behavioral responses of employees (Ruepert et al., 2017; Ahmad et al., 2021), the exploration for innovation of employees has only emerged in some recent literature. Besides, while previous research has stated that CSR perception might have a positive influence on employee creativity (Hur et al., 2018), few studies have linked perceived ECSR to the innovative behavior of employees in China. This study addressed this gap in our work by adding new insights regarding the important link in employees perceiving ECSR to promoting their innovative behavior. We conceptualize the perceived ECSR as a stimulus to elicit the innovative behavior of employees, which also echoes previous research (e.g., Shin et al., 2017; Boan and Dedeolu, 2020) to emphasize how some of the stimulus factors of innovative behavior are rooted in the perception of employees of environmental management and environmentally responsible activities. Moreover, this study clarifies the utility of the extended S-O-R model that can include perceived ECSR as a stimulus and innovative behavior as an employee response in the context of environmental protection. Furthermore, our findings extend the work in previous studies (e.g., Hur et al., 2018) by establishing the theoretical connection between perceived ECSR and innovative behavior from the stimulus–organism–response perspective.

Second, this study sheds a new theoretical light on both ECSR and innovative behavior literature by identifying the mediation effects of organizational identification. Previous research has investigated how individual identification can impact innovative behavior (Litchfield et al., 2018), but the role of organizational identification in the relationship between the perceived ECSR and the innovative behavior of employees is neglected in the existing literature. Because previous empirical studies have shown that organizational identification is a cognitive process in which individuals’ perception will affect their behavior (Tian and Robertson, 2019; Cheema et al., 2020). Thus, this study investigated organizational identification plays a mediating role in the relationship between the perceived ECSR and the innovative behavior of employees. Based on the S-O-R model, we highlighted that perceived ECSR is an important stimulus to gain organizational identification when perception stimulus occurs during the organizational identification judgment process. Our findings also strongly support our argument that the mediation process is conducive to better understanding the internal cognitive process of the impact of perceived ECSR on the innovative behavior of employees. Specifically, the serial mediation process in our study means that there is a process that increases their organizational identification when they perceive their firm as environmentally responsible, and thereby improves the innovative behavior of employees. Additionally, our findings responded to the recent call made by Tian and Robertson (2019) to pay more attention to the individual-level analysis in the ECSR research by revealing how organizational identification can act as a mediating role in perceived ECSR – innovative behavior of employees relationship.

Third, our study contributes to a better explanation of boundary conditions under which the relationship between the perceived ECSR and the innovative behavior of employees be maximized. Although previous studies have highlighted the value of perceived ECSR (Ahmad et al., 2021), there has been little understanding of when perceived ECSR can promote the innovative behavior of employees in the context of environmental protection. This limited understanding is because previous studies neglect the contextual factors that condition the effectiveness of the ECSR perception of employees. We addressed this gap in our work by identifying the appropriate boundary conditions that help firms to increase the potential benefits of the innovative behavior of employees. Considering trust as a positive and an essential element in the work environment, we attempted to integrate the influence of organizational trust in our S-O-R model to identify whether perceived ECSR – organizational identification – employees’ innovative behavior relationship varies across organizational trust levels. In combination with previous studies that treated organizational trust to be antecedents to the behavioral responses of individuals, our findings indicate organizational trust can be viewed as the moderator in the S-O-R model, providing insightful implications for academia and expanding the prior studies (e.g., Jani and Han, 2015). Overall, this study provides a better understanding that increased organizational trust in employees moderates positively the strength of the mediated relationship between ECSR perception and innovative behavior based on the S-O-R model, by strengthening not only the relationship between perceived ECSR and organizational identification, but also the indirect effect of perceived ECSR on employees’ innovative behavior via organizational identification. Thus, this study extends the boundary conditions of the effect of corporate social responsibility. Our findings also highlight the important role of organizational trust plays in impacting employees’ attitudes and behaviors.

### Practical Implications

Our findings also provide important practical implications for managers. First, the results indicate that the innovative behavior of employees is affected by ECSR perception. When employees perceive their firms as environmentally responsible, they are more likely to generate innovative behaviors so that firms could obtain a competitive advantage through the enhanced employee environmental performance (Lee et al., 2018; Tian and Robertson, 2019). Therefore, we suggest that firms who are willing to reduce the pressure of environmental protection through the innovative behavior of employees ought to take measures to strengthen the ECSR perceptions of employees. For example, managers can increase ECSR perception by involving employees in their ECSR activities. Further, managers should share the information with employees, such as waste emission reduction, pollution reduction, product recycling, and effective outcomes feedback.

Second, considering the significant effect of perceived ECSR on the innovative behavior of employees throughout the mediation of organizational identification, this study suggests that increasing the organizational identification of employees in their firms could be beneficial for eliciting the innovative behavior of employees. Thus, managers should pay more attention to fostering the organizational identification of employees toward firms. For instance, managers can enhance the organizational identification of employees by implementing ECSR activities of their firm and showing such activities as consistent with the values, beliefs, and goals of the firm to the employees. The shared values, beliefs, and goals of firms can help employees understand how the businesses operations in the natural environment, helping them foster a sense of identity with the environmental behavior of firms and then improving innovative behavior among employees at the individual levels. Besides, managers also can establish a working environment in which employees work in cooperation rather than compete with each other. In addition, to increase organizational identification of employees, managers should provide regular training (e.g., organizational culture training) to employees with low levels of education.

Finally, this study suggests that organizational trust, as a moderator, can effectively enhance the impact of perceived ECSR on the innovative behavior of employees in an organization, which provides a managerial implication. Thus, increasing the organizational trust of employees toward their firms could be beneficial from both environmental and ethical/moral perspectives, and particularly for the innovative behavior of employees. In this respect, managers should cultivate and enhance the organizational trust of employees in the HR processes (e.g., recruitment, training, or incentive design; Roeck and Delobbe, 2012) to maximize the potential return of the perceptions of ECSR. In the HR processes, to improve organizational trust, managers should adopt an effective way of sharing information, which might be future ECSR strategies, environmental performance feedback, and other work-related issues. Managers should offer employees complete and reliable information at work, and express a sense of unity. Furthermore, managers need to put more effort into showing integrity, which is important for building long-term commitment and developing trust.

### Limitations and Future Research

Although it has made contributions, our research still has some limitations which should be solved in future research. First, this study only investigated employees from some types of industries (e.g., non-ferrous metals, power, coal, mining, and pharmaceuticals) in China. It is difficult to generalize other countries and cultures by only relying on the sample data from one country. Future research should focus on examining different countries, such as industries or firms in more developed countries, and compare the results with this study. Second, we only tested the influence of organizational identification and organizational trust on the link between the perceived ECSR and the innovative behavior of employees based on the S-O-R framework. But the relationship between the perceived ECSR and the innovative behavior of employees is highly complex. To fully examine this complex relationship, future research should identify additional contingency factors (e.g., firm visibility; Wu W. et al., 2020) based on different theoretical perspectives, such as stakeholder theory. Third, only the innovative behavior of employees was examined in our study. In this respect, person-organization fit means that individuals and organizations can have a positive interaction, which may have a direct influence on innovative behavior and have an indirect impact on innovative behavior under the influence of internal motivation (Vilela et al., 2008). The innovative behavior of employees can be divided into two dimensions: idea generation and idea implementation (Scott and Bruce, 1994; Amabile and Pratt, 2016). Future research could further explore the effects of perceived ECSR on idea generation and idea implementation.

## Conclusion

In conclusion, this research sought to extend insights into the psychological mechanism between the perceived ECSR and the innovative behavior of employees using the S-O-R model. Our findings suggest that perceived ECSR positively affects organizational identification, which in turn are expected to influence the innovative behavior of employees. Further, our study extends the previous theory to confirm that organizational trust strengthens the effect of perceived ECSR on organizational identification and the effect of organizational identification on the innovative behavior of employees. We hope that this research has taken an important step toward the development of ECSR theory by explaining the effects of perceived ECSR on the innovative behavior of employees.

## Data Availability Statement

The raw data supporting the conclusions of this article will be made available by the authors, without undue reservation.

## Ethics Statement

The studies involving human participants were reviewed and approved by the institutional review board of Harbin Institute of Technology of China. The patients/participants provided their written informed consent to participate in this study.

## Author Contributions

WW and LY contributed to the conceptualization and design of the study. LY wrote the original draft. HL contributed to analyzing. TZ contributed to the review and editing of the manuscript. All authors contributed to the article and approved the submitted version.

## Conflict of Interest

The authors declare that the research was conducted in the absence of any commercial or financial relationships that could be construed as a potential conflict of interest.

## Publisher’s Note

All claims expressed in this article are solely those of the authors and do not necessarily represent those of their affiliated organizations, or those of the publisher, the editors and the reviewers. Any product that may be evaluated in this article, or claim that may be made by its manufacturer, is not guaranteed or endorsed by the publisher.

## References

[B1] Afsar B. Cheema S. Javed F. (2018). Activating employee’s pro-environmental behaviors: the role of CSR, organizational identification, and environmentally specific servant leadership. *Corp. Soc. Responsib. Environ. Manag.* 25 904–911. 10.1002/csr.1506

[B2] Ahmad N. Ullah Z. Arshad M. Z. Kamran H. Scholz M. Han H. (2021). Relationship between corporate social responsibility at the micro-level and environmental performance: the mediating role of employee pro-environmental behavior and the moderating role of gender. *Sustain. Prod. Consump.* 27 1138–1148. 10.1016/j.spc.2021.02.034

[B3] Ahmed M. Sun Z. Raza S. Qureshi M. Yousufi S. (2020). Impact of CSR and environmental triggers on employee green behavior: the mediating effect of employee well-being. *Corp. Soc. Responsib. Environ. Manag.* 27 2225–2239. 10.1002/csr.1960

[B4] Alfes K. Shantz A. Truss C. (2012). The link between perceived HRM practices, performance and well-being: the moderating effect of trust in the employer. *Hum. Resour. Manage. J.* 22 409–427. 10.1111/1748-8583.12005

[B5] Amabile T. M. Pratt M. G. (2016). The dynamic componential model of creativity and innovation in organizations: making progress, making meaning. *Res. Organ. Behav.* 36 157–183. 10.1016/j.riob.2016.10.001

[B6] Archimi C. S. Reynaud E. Yasin H. M. Bhatti Z. A. (2018). How perceived corporate social responsibility affects employee cynicism: the mediating role of organizational trust. *J. Bus. Ethics.* 151 907–921. 10.1007/s10551-018-3882-6

[B7] Ashforth B. E. Harrison S. H. Corley K. G. (2008). Identification in organizations: an examination of four fundamental questions. *J. Manage.* 34 325–374. 10.1177/0149206308316059

[B8] Ashforth B. E. Mael F. (1989). Social identity theory and the organization. *Acad. Manage. Rev.* 14, 20–39. 10.5465/AMR.1989.4278999

[B9] Bak H. (2020). Supervisor feedback and innovative work behavior: the mediating roles of trust in supervisor and affective commitment. *Front. Psychol.* 11:559160. 10.3389/fpsyg.2020.559160 33041923 PMC7526520

[B10] Barba-Sánchez V. Atienza-Sahuquillo C. (2017). Entrepreneurial motivation and self-employment: evidence from expectancy theory. *Int. Entrep. Manage. J.* 37 19–34. 10.1007/s11365-017-0441-z

[B11] Baughn C. C. Bodie N. Mcintosh J. C. (2007). Corporate social and environmental responsibility in asian countries and other geographical regions. *Corp. Soc. Responsib. Environ. Manag.* 14 189–205. 10.1002/csr.160

[B12] Bigne E. Chatzipanagiotou K. Ruiz C. (2020). Pictorial content, sequence of conflicting online reviews and consumer decision-making: the stimulus-organism-response model revisited. *J. Bus. Res.* 115 403–416. 10.1016/j.jbusres.2019.11.031

[B13] Boan E. Dedeolu B. B. (2020). Hotel employees’ corporate social responsibility perception and organizational citizenship behavior: perceived external prestige and pride in organization as serial mediators. *Corp. Soc. Responsib. Environ. Manag.* 27 2342–2353. 10.1002/csr.1996

[B14] Brammer S. He H. Mellahi K. (2015). Corporate social responsibility, employee organizational identification, and creative effort: the moderating impact of corporate ability. *Group Organ. Manage.* 40 323–352. 10.1177/1059601114562246

[B15] Castro-Gonzalez S. Bande B. Kimura T. (2019). How and when corporate social responsibility affects salespeople’s organizational citizenship behaviors? The moderating role of ethics and justice. *Corp. Soc. Responsib. Environ. Manag.* 26 548–558. 10.1002/csr.1700

[B16] Chathoth P. K. Mak B. Sim J. Jauhari V. Manaktola K. (2011). Assessing dimensions of organizational trust across cultures: a comparative analysis of U.S. and Indian full service hotels. *Int. J. Hosp. Manage.* 30 233–242. 10.1016/j.ijhm.2010.09.004

[B17] Cheema S. Afsar B. Javed F. (2020). Employees’ corporate social responsibility perceptions and organizational citizenship behaviors for the environment: the mediating roles of organizational identification and environmental orientation fit. *Corp. Soc. Responsib. Environ. Manag.* 27 9–21. 10.1002/csr.1769

[B18] Chuang S.-P. Huang S.-J. (2018). The effect of environmental corporate social responsibility on environmental performance and business competitiveness: the mediation of green information technology capital. *J. Bus. Ethics.* 150 991–1009. 10.1007/s10551-016-3167-x

[B19] Comrey A. L. (1988). Factor-analytic methods of scale development in personality and clinical psychology. *J. Consult. Clin. Psychol*. 56 754–761. 10.1037/0022-006X.56.5.754 3057010

[B20] Cordeiro J. J. Tewari M. (2014). Firm characteristics, industry context, and investor reactions to environmental CSR: a stakeholder theory approach. *J. Bus. Ethics.* 130 833–849. 10.1007/s10551-014-2115-x

[B21] De Roeck K. El Akremi A. Swaen V. (2016). Consistency matters! How and when does corporate social responsibility affect employees’ organizational identification? *J. Manage. Stud.* 53 1141–1168. 10.1111/joms.12216

[B22] Dirks K. T. Ferrin D. L. (2002). Trust in leadership: meta-analytic findings and implications for research and practice. *J. Appl. Psychol.* 87 611–628. 10.1037//0021-9010.87.4.611 12184567

[B23] Dukerich J. M. Golden B. R. Shortell S. M. (2002). Beauty is in the eye of the beholder: the impact of organizational identification, identity, and image on the cooperative behaviors of physicians. *Adm. Sci. Q.* 47 507–533. 10.2307/3094849

[B24] Dutton J. E. Dukerich J. M. Harquail C. V. (1994). Organizational images and member identification. *Adm. Sci. Q.* 39 239–263. 10.2307/2393235

[B25] Eroglu S. A. Machleit K. A. Davis L. M. (2001). Atmospheric qualities of online retailing - a conceptual model and implications. *J. Bus. Res.* 54 177–184. 10.1016/S0148-2963(99)00087-9

[B26] Ertürk A. (2010). Exploring predictors of organizational identification: moderating role of trust on the associations between empowerment, organizational support, and identification. *Eur. J. Work Organ. Psychol.* 19 409–441. 10.1080/13594320902834149

[B27] Ertürk A. Vurgun L. (2015). Retention of it professionals: examining the influence of empowerment, social exchange, and trust. *J. Bus. Res.* 68 34–46. 10.1016/j.jbusres.2014.05.010

[B28] Farooq O. Payaud M. Merunka D. Valette-Florence P. (2014). The impact of corporate social responsibility on organizational commitment: exploring multiple mediation mechanisms. *J. Bus. Ethics.* 125 563–580. 10.1007/s10551-013-1928-3

[B29] Farooq O. Rupp D. Farooq M. (2017). The multiple pathways therough which internal and external corporate social responsibility influence organizational identification and multifoci outcomes: the moderating role of cultural and social orientations. *Acad. Manage. J.* 60 954–985. 10.5465/amj.2014.0849

[B30] Farrington T. Curran R. Gori K. O’Gorman K. D. Queenan C. J. (2017). Corporate social responsibility: reviewed, rated, revised. *Int. J. Contemp. Hosp. Manage.* 29 30–47. 10.1108/IJCHM-05-2015-0236

[B31] Farrukh M. Ansari N. Y. Raza A. Meng F. Wang H. (2021). High-performance work practices do much, but H.E.R.O does more: an empirical investigation of employees’ innovative behavior from the hospitality industry. *Eur. J. Innov. Manage [Online ahead of print]* 10.1108/EJIM-11-2020-0448

[B32] Ferdous A. S. Polonsky M. Bednall D. H. B. (2021). Internal communication and the development of customer-oriented behavior among frontline employees. *Eur. J. Marketing* 47 422–430. 10.1108/EJM-10-2019-0750

[B33] Flammer C. (2013). Corporate social responsibility and shareholder reaction: the environmental awareness of investors. *Acad. Manage. J.* 56 758–781. 10.5465/amj.2011.0744

[B34] Forcadell F. J. Úbedac F. Aracil E. (2021). Effects of environmental corporate social responsibility on innovativeness of spanish industrial SMEs. *Technol. Forecast. Soc. Change* 162:120355. 10.1016/j.techfore.2020.120355

[B35] Fornell C. Larcker D. F. (1981). Evaluating structural equation models with unobservable variables and measurement error. *J. Marketing Res.* 48 39–50. 10.1177/002224378101800312

[B36] Fuller C. M. Simmering M. J. Atinc G. Atinc Y. Babin B. J. (2016). Common methods variance detection in business research. *J. Bus. Res.* 69 3192–3198. 10.1016/j.jbusres.2015.12.008

[B37] Galbreath J. (2019). Drivers of green innovations: the impact of export intensity, women leaders, and absorptive capacity. *J. Bus. Ethics.* 158 47–61. 10.1007/s10551-017-3715-z

[B38] Glavas A. (2016). Corporate social responsibility and organizational psychology: an integrative review. *Front. Psychol.* 7:144. 10.3389/fpsyg.2016.00144 26909055 PMC4754563

[B39] Han B. (2021). Research on the influence of technological innovation on carbon productivity and countermeasures in China. *Environ. Sci. Pollut. Res*. 28 16880–16894. 10.1007/s11356-020-11890-x 33392990

[B40] Hansen S. Dunford B. Boss A. Boss R. Angermeier I. (2011). Corporate social responsibility and the benefits of employee trust: a cross-disciplinary perspective. *J. Bus. Ethics.* 102 29–45. 10.1007/s10551-011-0903-0

[B41] Harman H. H. (1961). Modern factor analysis. *J. Amer. Stat. Assoc.* 56 444–446. 10.2307/2282293

[B42] Hayes A. (2013). *Introduction to Mediation, Moderation, and Conditional Process Analysis: A Regression-Based Approach*, 1st Edn. New York, NY: Guilford Press.

[B43] Hofman P. S. Newman A. (2014). The impact of perceived corporate social responsibility on organizational commitment and the moderating role of collectivism and masculinity: evidence from China. *Int. J. Hum. Resour. Manage.* 25 631–652. 10.1080/09585192.2013.792861

[B44] Hosmer L. T. (1995). Trust: the connecting link between organizational theory and philosophical ethics. *Acad. Manage. Rev.* 20 379–403. 10.2307/258851

[B45] Huang Y. S. Wei S. Ang T. (2021). The role of customer perceived ethicality in explaining the impact of incivility among employees on customer unethical behavior and customer citizenship behavior. *J. Bus. Ethics.* 10.1007/s10551-020-04698-9

[B46] Hur W. M. Moon T.-W. Ko S.-H. (2018). How employees’ perceptions of CSR increase employee creativity: mediating mechanisms of compassion at work intrinsic motivation. *J. Bus. Ethics* 153 629–644. 10.1007/s10551-016-3321-5

[B47] Ilkhanizadeh S. Karatepe O. M. (2017). An examination of the consequences of corporate social responsibility in the airline industry: work engagement, career satisfaction, and voice behavior. *J. Air Transp. Manage.* 59 8–17. 10.1016/j.jairtraman.2016.11.002

[B48] Islam T. Ahmed I. Ali G. Sadiq T. (2016). Behavioral and psychological consequences of corporate social responsibility: need of the time. *Soc. Resp. J.* 12 307–320. 10.1108/srj-04-2015-0053

[B49] Jacoby J. (2002). Stimulus-organism-response reconsidered: an evolutionary step in modeling (consumer) behavior. *J. Consum. Psychol.* 12 51–57. 10.1207/S15327663JCP1201_05

[B50] Jang S. C. Namkung Y. (2009). Perceived quality, emotions, and behavioral intentions: application of an extended mehrabian–russell model to restaurants. *J. Bus. Res.* 62 451–460. 10.1016/j.jbusres.2008.01.038

[B51] Jani D. Han H. (2015). Influence of environmental stimuli on hotel customer emotional loyalty response: testing the moderating effect of the big five personality factors. *Int. J. Hosp. Manage.* 44 48–57. 10.1016/j.ijhm.2014.10.006

[B52] Javed B. Khan A. K. Arjoon S. Mashkoor M. Haque A. U. (2020). Openness to experience, ethical leadership, and innovative work behavior. *J. Creative Behav.* 54 211–223. 10.1002/jocb.360

[B53] Kim B. Lee S. Kang K. H. (2018). The moderating role of CEO narcissism on the relationship between uncertainty avoidance and CSR. *Tourism Manage.* 67 203–213. 10.1016/j.tourman.2018.01.018

[B54] Kim B. J. Kim M. J. Kim T. H. (2021). “The power of ethical leadership”: the influence of corporate social responsibility on creativity, the mediating function of psychological safety, and the moderating role of ethical leadership. *Int. J. Env. Res. Public Health* 18:2968. 10.3390/ijerph18062968 33799360 PMC7998312

[B55] Kim H. Park K. Ryu D. (2017). Corporate environmental responsibility: a legal origins perspective. *J. Bus. Ethics* 140 381–402. 10.1007/s10551-015-2641-1

[B56] Kim H. R. Lee M. Lee H. T. Kim N. M. (2010). Corporate social responsibility and employee–company identification. *J. Bus. Ethics* 95 557–569. 10.1007/s10551-010-0440-2

[B57] Kim J. Lennon S. J. (2013). Effects of reputation and website quality on online consumers’ emotion, perceived risk and purchase intention. *J. Res. Interact. Marketing* 7 33–56. 10.1108/17505931311316734

[B58] Kim J. Song H. J. Lee C. K. (2016). Effects of corporate social responsibility and internal marketing on organizational commitment and turnover intentions. *Int. J. Hosp. Manage.* 55 25–32. 10.1016/j.ijhm.2016.02.007

[B59] Korschun D. Bhattacharya C. B. Swain S. D. (2014). Corporate social responsibility, customer orientation, and the job performance of frontline employees. ESMTresearch working papers. *J. Marketing* 78 20–37. 10.2139/ssrn.1856475

[B60] Kwon K. Kim T. (2020). An integrative literature review of employee engagement and innovative behavior: revisiting the JD-R model. *Hum. Resour. Manage. R* 30:100704.

[B61] Lee C.-K. Song H.-J. Lee H.-M. Lee S. Bernhard B. J. (2013). The impact of CSR on casino employees’ organizational trust, job satisfaction, and customer orientation: an empirical examination of responsible gambling strategies. *Int. J. Hosp. Manage.* 33 406–415. 10.1016/j.ijhm.2012.10.011

[B62] Lee J. W. Kim Y. M. Kim Y. E. (2018). Antecedents of adopting corporate environmental responsibility and green practices. *J. Bus. Ethics.* 148 397–409. 10.1007/s10551-016-3024-y

[B63] Li M. J. Tao W. Q. Yan J. (2017). Review of methodologies and polices for evaluation of energy efficiency in high energy-consuming industry. *Appl. Energ*. 87 203–215. 10.1016/j.apenergy.2016.11.039

[B64] Li Z. Liao G. Albitar K. (2020). Does corporate environmental responsibility engagement affect firm value? The mediating role of corporate innovation. *Bus. Strateg. Environ.* 29 1045–1055. 10.1002/bse.2416

[B65] Lioui A. Sharma Z. (2012). Environmental corporate social responsibility and financial performance: disentangling direct and indirect effects. *Ecol. Econ.* 78 100–111. 10.1016/j.ecolecon.2012.04.004

[B66] Litchfield R. C. Karakitapolu-Aygün Z. Gumusluoglu L. Carter M. Hirst G. (2018). When team identity helps innovation and when it hurts: team identity and its relationship to team and cross-team innovative behavior. *J. Prod. Innov. Manage*. 35 350–366. 10.1111/jpim.12410

[B67] Liu Y. Wang W. Chen D. (2019). Linking ambidextrous organizational culture to innovative behavior: a moderated mediation model of psychological empowerment and transformational leadership. *Front. Psychol.* 10:2192. 10.3389/fpsyg.2019.02192 31681063 PMC6798063

[B68] Madjar N. Greenberg E. Chen Z. (2011). Factors for radical creativity, incremental creativity, and routine, noncreative performance. *J. Appl. Psychol.* 96 730–743. 10.1037/a0022416 21319879

[B69] Mael F. Ashforth B. E. (1992). Alumni and their alma mater: a partial test of the reformulated model of organizational identification. *J. Organ. Behav.* 13 103–123. 10.1002/job.4030130202

[B70] Mazurkiewicz P. (2004). *Corporate Environmental Responsibility: Is A Common CSR Framework Possible. World Bank.* Availalble online at: http://siteresources.worldbank.org/EXTDEVCOMSUSDEVT/Resources/csrframework.pdf (accessed Octobe 20, 2014).

[B71] Mehrabian A. Russell J. A. (1974). *An Approach to Environmental Psychology.* Cambridge: M.I.T. Press.

[B72] Miao Q. Newman A. Schwarz G. Cooper B. (2018). How leadership and public service motivation enhance innovative behavior. *Pub. Admin. Rev.* 78 71–81. 10.1111/puar.12839

[B73] National Bureau of Statistics of the People’s Republic of China (2011). *The Statistical Report on National Economic and Social Development*. Available online at: http://www.stats.gov.cn/tjsj/tjgb/ndtjgb/qgndtjgb/201202/t20120222_30026.html

[B74] Orazalin N. (2020). Do board sustainability committees contribute to corporate environmental and social performance? The mediating role of corporate social responsibility strategy. *Bus. Strateg. Environ.* 29 140–153. 10.1002/bse.2354

[B75] Parker C. P. Baltes B. B. Young S. A. Huff J. W. Altmann R. A. LaCost H. A. (2003). Relationships between psychological climate perceptions and work outcomes: a meta-analytic review. *J. Organ. Behav.* 24 389–416. 10.1002/job.198

[B76] Perry R. W. Mankin L. D. (2007). Organizational trust, trust in the chief executive and work satisfaction. *Public Pers. Manage.* 36 165–179. 10.1177/009102600703600205

[B77] Podsakoff P. M. Mackenzie S. B. Lee J. Y. Podsakoff N. P. (2003). Common method biases in behavioral research: a critical review of the literature and recommended remedies. *J. Appl. Psychol.* 88 879–903. 10.1037/0021-9010.88.5.879 14516251

[B78] Pučėtaitė R. Lämsä A.-M. (2008). Developing organizational trust through advancement of employees’ work ethic in a post-socialist context. *J. Bus. Ethics* 82 325–337. 10.1007/s10551-008-9922-x

[B79] Rahman N. Post C. (2012). Measurement issues in environmental corporate social responsibility (ECSR): toward a transparent, reliable, and construct valid instrument. *J. Bus. Ethics* 105 307–319. 10.1007/s10551-011-0967-x

[B80] Rela I. Awang A. Ramli Z. Sum S. Meisanti M. (2021). Effects of environmental corporate social responsibility on environmentalwell-beingperception and the mediation role of community resilience. *Corp. Soc. Responsib. Environ. Manag.* 27 2176–2187. 10.1002/csr.1956

[B81] Reynolds N. Diamantopoulos A. Schlegelmilch B. (1993). Pretesting in questionnaire design: a review of the literature and suggestions for further research. *J. Mark. Res. Soc.* 35 171–182. 10.1007/BF01065358

[B82] Roeck K. D. Delobbe N. (2012). Do environmental CSR initiatives serve organizations’ legitimacy in the oil industry? Exploring employees’ reactions through organizational identification theory. *J. Bus. Ethics.* 110 397–412. 10.1007/s10551-012-1489-x

[B83] Roeck K. D. Farooq O. (2017). Corporate social responsibility and ethical leadership: investigating their interactive effect on employees’ socially responsible behaviors. *J. Bus. Ethics* 151 923–939. 10.1007/s10551-017-3656-6

[B84] Ruepert A. M. Keizer K. Steg L. (2017). The relationship between corporate environmental responsibility, employees’ biospheric values and pro-environmental behaviour at work. *J. Environ. Psychol.* 54 65–78. 10.1016/j.jenvp.2017.10.006

[B85] Sánchez J. Callarisa L. Rodríguez R. Moliner M. A. (2006). Perceived value of the purchase of a tourism product. *Tourism Manage.* 27 394–409. 10.1016/j.tourman.2004.11.007

[B86] Scott S. G. Bruce R. A. (1994). Determinants of innovative behavior: a path model of individual innovation in the workplace. *Acad. Manage. J.* 37 580–607. 10.2307/256701

[B87] Shah S. Cheema S. Al-Ghazali B. Ali M. Rafiq N. (2020). Perceived corporate social responsibility and pro-environmental behaviors: the role of organizational identification and coworker pro-environmental advocacy. *Corp. Soc. Responsib. Environ. Manag.* 28 366–377. 10.1002/csr.2054

[B88] Shah S. Sarfraz M. Ivascu L. (2021). Assessing the interrelationship corporate environmental responsibility, innovative strategies, cognitive and hierarchical ceo: a stakeholder theory perspective. *Corp. Soc. Responsib. Environ. Manag.* 28 457–473. 10.1002/csr.2061

[B89] Shin S. J. Yuan F. Zhou J. (2017). When perceived innovation job requirement increases employee innovative behavior: a sensemaking perspective. *J. Organ. Behav.* 38 68–86. 10.1002/job.2111

[B90] Song W. H. Ren S. C. Yu J. (2019). Bridging the gap between corporate social responsibility and new green product success: the role of green organizational identity. *Bus. Strategy Environ.* 28, 88–97. 10.1002/bse.2205

[B91] Spector P. E. (1994). Using self-report questionnaires in ob research: a comment on the use of a controversial method. *J. Organ. Behav.* 15 385–392. 10.1002/job.4030150503

[B92] Su L. Swanson S. R. (2019). Perceived corporate social responsibility’s impact on the well-being and supportive green behaviors of hotel employees: the mediating role of the employee-corporate relationship. *Tourism Manage.* 72 437–450. 10.1016/j.tourman.2019.01.009

[B93] Tang Y. Shao Y. Chen Y. Ma Y. (2021). How to keep sustainable development between enterprises and employees? Evaluating the impact of person–organization fit and person–job fit on innovative behavior. *Front. Psychol.* 12:653534. 10.3389/fpsyg.2021.653534 33995213 PMC8119782

[B94] Tang Z. Warkentin M. Le W. (2019). Understanding employees’ energy saving behavior from the perspective of stimulus-organism-responses. *Resour. Conserv. Recycl.* 140 216–223. 10.1016/j.resconrec.2018.09.030

[B95] Taniguchi H. Marshall G. (2018). Trust, political orientation, and environmental behavior. *Env. Polit.* 27 385–410. 10.1080/09644016.2018.1425275

[B96] Thomas W. H. (2015). The incremental validity of organizational commitment, organizational trust, and organizational identification. *J. Vocat. Behav.* 88 154–163. 10.1016/j.jvb.2015.03.003

[B97] Tian Q. Robertson J. L. (2019). How and when does perceived CSR affect employees’ engagement in voluntary pro-environmental behavior? *J. Bus. Ethics.* 155 399–412. 10.1007/s10551-017-3497-3

[B98] Turker D. (2009). Measuring corporate social responsibility: a scale development study. *J. Bus. Ethics.* 85 411–427. 10.1007/s10551-008-9780-6

[B99] Van Dick R. Crawshaw J. R. Karpf S. Schuh S. C. Zhang X. A. (2020). Identity, importance, and their roles in how corporate social responsibility affects workplace attitudes and behavior. *J. Bus. Psychol.* 35 159–169. 10.1007/s10869-019-09619-w

[B100] Vilela B. B. González J. A. V. Ferrín P. F. (2008). Person–organization fit, ocb and performance appraisal: evidence from matched supervisor–salesperson data set in a spanish context. *Ind. Market. Manage.* 37 1005–1019. 10.1016/j.indmarman.2007.11.004

[B101] Vlachos P. A. Theotokis A. Panagopoulos N. G. (2010). Sales force reactions to corporate social responsibility: attributions, outcomes, and the mediating role of organizational trust. *Ind. Market. Manage.* 39 1207–1218. 10.1016/j.indmarman.2010.02.004

[B102] Walker K. Ni N. Huo W. (2014). Is the red dragon green? An examination of the antecedents and consequences of environmental proactivity in China. *J. Bus. Ethics* 125 27–43. 10.1007/s10551-013-1903-z

[B103] Wang H. Chen M. Li X. (2021). Moderating multiple mediation model of the impact of inclusive leadership on employee innovative behavior. *Front. Psychol.* 12:666477. 10.3389/fpsyg.2021.666477 34456787 PMC8385277

[B104] Wei Z. Hao S. Zhou K. Z. Li J. J. (2017). How does environmental corporate social responsibility matter in a dysfunctional institutional environment? Evidence from China. *J. Bus. Ethics* 140 209–223. 10.1007/s10551-015-2704-3

[B105] Wu C.-H. Jong J. D. Raasch C. Poldervaart S. (2020). Work process-related lead userness as an antecedent of innovative behavior and user innovation in organizations. *Res. Pol.* 49:103986. 10.1016/j.respol.2020.103986

[B106] Wu W. Liang Z. Zhang Q. (2020). Effects of corporate environmental responsibility strength and concern on innovation performance: the moderating role of firm visibility. *Corp. Soc. Responsib. Environ. Manag.* 27 1487–1497. 10.1002/csr.1902

[B107] Xu F. Wang Y. (2019). Enhancing employee innovation through customer engagement: the role of customer interactivity, employee affect, and motivations. *J. Hosp. Tourism Res.* 44 351–376. 10.1177/1096348019893043

[B108] Xu X. Zeng S. Chen H. (2018). Signaling good by doing good: how does environmental corporate social responsibility affect international expansion? *Bus. Strategy Environ.* 27 946–959. 10.1002/bse.2044

[B109] Yuan F. Woodman R. W. (2010). Innovative behavior in the workplace: the role of performance and image outcome expectations. *Acad. Manage. J.* 53 323–342. 10.5465/AMJ.2010.49388995

[B110] Yuan Y. (2021). Leader–employee congruence in humor and innovative behavior: the moderating role of dynamic tenure. *Front. Psychol.* 12:579551. 10.3389/fpsyg.2021.579551 33746818 PMC7973860

[B111] Zhang Y. Ouyang Z. (2021). Doing well by doing good: how corporate environmental responsibility influences corporate financial performance. *Corp. Soc. Responsib. Environ. Manag.* 28 54–63. 10.1002/csr.2031

[B112] Zhang Y. J. Liu J. Y. (2019). Does carbon emissions trading affect the financial performance of high energy-consuming firms in China? *Nat. Hazards* 95 91–111. 10.1007/s11069-018-3434-5

[B113] Zhou Z. Luo B. N. Tang L. P. (2018). Corporate social responsibility excites ‘exponential’ positive employee engagement: the matthew effect in CSR and sustainable policy: CSR exponentially reaps intangible rewards. *Corp. Soc. Responsib. Environ. Manag.* 25 339–354. 10.1002/csr.1464

